# Closing the hospice gap: the value of home-based medical care

**DOI:** 10.1093/haschl/qxag251

**Published:** 2026-09-17

**Authors:** Katherine Ornstein, Bruce Leff, Maggie Salinger, Jennifer M Reckrey, Bian Liu, Zainab Toteh Osakwe, Martha Abshire Saylor, Arushi Arora, Melissa Aldridge, Krista Harrison, Abraham A Brody, Christine S Ritchie

**Affiliations:** Center for Equity in Aging, School of Nursing, Johns Hopkins University, Baltimore, MD 21205, United States; Division of Geriatric Medicine and Gerontology, School of Medicine, Johns Hopkins University, Baltimore, MD 21224, United States; Department of Medicine, Warren Alpert Medical School at Brown University, Providence, RI 02903, United States; Division of Geriatric Medicine and Palliative Care, Department of Medicine, NYU Grossman School of Medicine, New York, NY 10017, United States; Department of Population Health Science and Policy, Icahn School of Medicine at Mount Sinai, New York, NY 10029, United States; College of Nursing and Public Health, Adelphi University, Garden City, NY 11530, United States; Center for Equity in Aging, School of Nursing, Johns Hopkins University, Baltimore, MD 21205, United States; Department of Geriatrics and Palliative Medicine, Icahn School of Medicine at Mount Sinai, New York, NY 10029, United States; Department of Geriatrics and Palliative Medicine, Icahn School of Medicine at Mount Sinai, New York, NY 10029, United States; Division of Geriatrics and Philip R. Lee Institute for Health Policy Studies, School of Medicine, University of California San Francisco, San Francisco, CA 94158, United States; Rory Meyers College of Nursing, New York University, New York, NY 10010, United States; Center for Optimal Aging and Serious Illness Research, Massachusetts General Hospital, Boston, MA 02114, United States

**Keywords:** hospice, home-based medical care, older adults

## Abstract

**Introduction:**

Home-based medical care (HBMC), longitudinal medical care provided in a patient's home led by physicians or advanced practice providers, addresses complex medical and social needs of seriously ill older adults, particularly those with dementia. We examined whether HBMC use is associated with hospice enrollment and length of stay and whether this varies by dementia status.

**Methods:**

We analyzed data from 100% fee-for-service Medicare decedents in 2019, who were eligible for HBMC prior to death. Models used inverse probability weighting, adjusting for clinical, demographic, and regional characteristics.

**Results:**

HBMC users had greater odds of hospice use (odds ratio: 1.34; 95% CI: 1.33-1.35) compared with nonusers, regardless of dementia status. For hospice enrollees, HBMC use minimized both ultra-short (<3 days) and prolonged (>180 days) stays, with a stronger reduction in short stays for patients with dementia.

**Conclusion:**

As more older adults age in the community and hospice services expand to support patients with complex illnesses such as dementia, HBMC may offer a valuable strategy for effectively navigating prognosis uncertainty and the complexities of hospice eligibility requirements and enrollment.

## Introduction

As the locus of long-term care shifts away from nursing homes,^1^ older adults with serious illness, including dementia, are increasingly living and dying at home with the support of family caregivers, paid caregivers, and staff at community-based facilities (eg, assisted living). Homebound older adults are heterogeneous, requiring different caregiving support,^2^ have complex clinical and social needs, high health care spending,^3^ high symptom burden,^4^ and high mortality^5^ and may experience difficulty accessing traditional office-based primary care.^6^

The shift of medical care to the home encompasses several care delivery models including home hospice, skilled home health care and home-based medical care (HBMC).^7^ Clinical HBMC (ie, house calls or home-based primary care) provides longitudinal medical care in the home (including assisted-living facilities) led by physicians or advanced practice providers. Home-based medical care serves patients with serious illness and high mortality and can serve as a substitute for office-based care.^8^ It often includes interdisciplinary team care and occurs in coordination with other home-based delivery models, such as skilled home health care. Home-based medical care has been associated with reductions in hospital admissions, emergency department visits, hospital length of stays, and long-term care admissions and notably provided critical support during the COVID-19 emergency to vulnerable older adults, including those with dementia.^9^ Although increasing, particularly in assisted-living and other domiciliary settings,^10^ HBMC is not widely accessible in the United States, with variation based on region and rurality.^11,12^

Hospice use shows consistent benefits (eg, reduced cost, improved perceived quality) for persons with serious illnesses, including for persons with dementia, and is widely considered a marker of high-quality end-of-life care.^13^ In fact, while hospice was originally developed for persons with end-stage cancer, dementia is now the most common hospice enrollment diagnosis.^14^ Persons with dementia often have increasing care needs at the end of life and fewer options for in-home support.^15,16^ Yet, there remains extensive variation in quality and utilization of hospice care among persons with dementia, particularly related to length of stay. Average hospice length of stay is approximately 17 days nationwide, but many people with dementia experience either long hospice stays, which can put them at risk of loss of hospice benefit through live discharge which can be stressful for patients and families,^17^ or a hospice stay that is too short to allow patients and families to benefit from hospice care.^18-20^ There are numerous factors that contribute to both use of hospice and variation in duration of hospice care, including patient, caregiver, geographic, and provider factors (eg, hospice for-profit vs not-for-profit status).^19^ Although patients using HBMC have high mortality rates, high symptom burden, and frequently voice a preference for in-home, non–hospital-based care at the end of life, there has been limited research to examine how HBMC use may or may not facilitate the use and timing of hospice care.

Therefore, it is vital to understand if and how HBMC affects the end-of-life experience, particularly hospice use. This study hypothesizes that, among individuals with dementia or other serious illnesses, HBMC can facilitate the timely transition to hospice.

## Data and methods

### Data source and sample derivation

We conducted a retrospective cohort study of US Medicare fee-for-service (FFS) beneficiaries who died in 2019, examining HBMC receipt in the 2 years prior to death. These were the most recent data available prior to the COVID-19 pandemic (during which home-based care delivery, including hospice use, was disrupted).^21^

At the beneficiary level, we linked sociodemographic characteristics and residential geography from the Master Beneficiary Summary File and hospital utilization from the inpatient Medicare Provider and Analysis Review (MedPAR) file, HBMC use from Part B, and hospice enrollment status from hospice claims. We also used beneficiary zip codes for linkage with area-level characteristics.

Our cohort comprised 100% of Medicare decedents in 2019 who (1) were aged 67 years or older at death, (2) were continuously enrolled in FFS Medicare 2018–2019, and (3) met eligibility criteria for HBMC 366 to 730 days prior to death (sample derivation is depicted in Appendix Figure S1). Eligibility criteria for HBMC have been defined through our prior work involving a modified Delphi process and construct validation.^22^ Briefly, the available HBMC criteria in claims data include presence of serious illness including dementia,^23^ eligibility for the Centers for Medicare and Medicaid Services Innovation Center Independence at Home Demonstration Program (≥2 chronic conditions, ≥2 functional dependencies, a nonelective hospitalization followed by acute or subacute rehab services in the last 12 months,^24^ and/or documentation of pressure ulcers in home setting). These criteria were determined in the 366 to 730 days prior to death to ensure HBMC eligibility temporally preceded the period in which we examined HBMC utilization (ie, the year prior to death).

### Measures

#### Hospice use and length of hospice care

The primary outcome was any hospice use (either at home or inpatient) during the last year of life. The secondary outcome was duration of hospice care in days, which was based on total length of stay, including across multiple stays (limited to 5 stays per year). We categorized hospice length of stay by mutually exclusive periods of hospice enrollment, based on the number of days before death that enrollment occurred, as follows: 1 to 3 (exceedingly short), 4 to 17, 18 to 60, 61 to 180, and 181 days or more (prolonged). We used clinically and policy-relevant cut points to create meaningful categories; for instance, 3 days is both the approximate time it takes to effectively set up hospice services and is the cut point for the Centers for Medicare and Medicaid Services’ last 3 days of life measure, 17 days is the national median length of stay, 60 days is the threshold for change in payment, and 180 days requires re-certification. Of note, if individuals were enrolled and disenrolled in hospice on the same day, they did not count as having any hospice care. If individuals enrolled and died on the same day, they were counted as having 1 day of hospice.

#### Use and timing of HBMC

The main independent variable was a binary indicator of HBMC use in the last year of life. Based on our previous work, we used Medicare Part B data to identify Healthcare Common Procedure Coding System codes to identify HBMC visits that occurred both in beneficiaries’ homes (99341-50) and in domiciliary settings—for example, assisted living (99324-8 and 99334-7)—in the 12 months before death.^7,10^ We counted a maximum of 1 visit per day and used the 2 or more HBMC visits threshold for defining HBMC to distinguish longitudinal HBMC enrollment from incidental or one-time home visits in the 12 months before death.^25^ Regardless of total number of HBMC visits a patient ultimately received, we required at least 1 HBMC visit to occur 1 to 90 days prior to hospice initiation. This 90-day window was selected to ensure temporality while aligning with the typical HBMC visit frequency of every 2 to 3 months. This conservative window minimizes the likelihood of overestimating the association between HBMC and hospice enrollment (note: HBMC can continue beyond hospice initiation and visits typically occur every 1–3 months). Individuals who had only 1 HBMC visit in the year before death, who did not have at least 1 HBMC visit prior to their first hospice visit, whose first HBMC visit was more than 90 days prior to hospice, or had missing covariates were excluded from analyses (6.5% of decedents eligible for HBMC) (Appendix Figure S1).

#### Covariates

To account for possible confounding, we selected sociodemographic, clinical, and area-level characteristics as covariates a priori based on previous research on factors^26^ associated with HBMC and hospice use and data availability. Sociodemographic factors included age at death, sex, and race/ethnicity. Dementia status was determined via the Bynum-standard algorithm^27^ up to 365 days prior to date of death. Clinical indicators were the Charlson Comorbidity Index to measure chronic illness burden, a claims-based frailty index,^28^ and a binary indicator of 2 or more hospitalizations, with all variables assessed 366 to 730 days before death to ensure temporality in study design. Finally, we examined the following area-level factors: census region, rural vs urban county classification,^29^ 2019 Social Deprivation Index to identify disadvantaged neighborhoods,^30^ and 2019 indicator of HBMC provider density.^31^

### Statistical analysis

First, we characterized the overall sample based on use of HBMC. We then used inverse probability weighting (IPW) to balance patient sociodemographic, clinical, and area-level characteristics between those with and without HBMC use, assessing balance with standardized mean differences (SMDs). An absolute SMD of less than 0.1 was considered to represent a good balance between the groups.^32^

We conducted logistic regression analyses to assess the association between HBMC use and the primary outcome of any hospice use. To examine the association of HBMC use with hospice length of stay, we performed a series of multinomial logistic regression analyses among hospice users. We defined the reference hospice duration category as 18 to 60 days (which included median length of stay in the sample). To understand whether HBMC use had differential associations with hospice utilization for beneficiaries with vs without dementia, we then performed these logistic and multinomial regression models (1) with an interaction term between dementia status and HBMC use and (2) stratified by dementia status. To protect against model misspecification, we also adjusted for all covariates in our regression models. All analyses were conducted using SAS Enterprise Guide version 8.

### Limitations

Our study limitations included the inability to adjust for unmeasured characteristics, such as care preferences, which are not available in our data. Although imperfect, IPW methodology is among the most rigorous tools available to compare groups using observational data and has been used in a wide range of studies to inform policy-relevant questions. While we attempted to address potential selection bias into HBMC by restricting the cohort to individuals who were clinically eligible based on claims data, confounding may still exist.

Further, the analysis is restricted to beneficiaries in FFS Medicare, and as Medicare Advantage (MA) enrollment continues to increase, the evolving role of MA plans will likely remain a critical factor influencing access to HBMC.^33^ The data, from 2018–2019, may not reflect more recent trends in care delivery. Additionally, HBMC is a heterogeneous intervention, and this variability could bias results toward the null, suggesting that our observed associations may be conservative. This study did not examine several factors that may also influence hospice use and patterns of care, including hospice ownership and affiliations between HBMC practices and hospice agencies. Furthermore, we could not account for assisted-living facility residence among non-HBMC users. As HBMC use continues to grow in congregate settings,^10^ more work will be needed to understand how HBMC and assisted-living facility care jointly work to facilitate hospice care. Finally, we did not assess hospice disenrollment rates, which have previously been found to be disproportionately high among people with dementia and racial and ethnic minority groups.^20^

## Results

Our final sample included 585 226 decedents who met eligibility for HBMC (mean age: 83.5 years; 56.9% female; and 54.8% with dementia). Of these, 13.4% (*n* = 78 716) used HBMC. In Table 1, we present unweighted characteristics of the sample of decedents by HBMC status. Briefly, compared with those who did not use HBMC, those who used HBMC were, on average, older, more often female, and more likely to have dementia and be frail. The HBMC users were more likely to live in urban areas, areas with greater density of HBMC providers.

**Table 1.** Patient characteristics and hospice use by HBMC use (unweighted).

	Non–HBMC users	HBMC users	Total
No. (%)	506 510 (86.6)	78 716 (13.4)	585 226
Hospice use^a^			
Any hospice use, No. (%)	302 369 (59.7)	55 692 (70.8)	358 051 (61.2)
Median hospice length of stay, d	20 (6-98)	28 (7-97)	21 (6-98)
Demographic characteristics			
Age, mean (SD), y	83.01 (8.7)	86.5 (8.0)	83.48 (8.7)
Female sex, No. (%)	282 660 (55.8)	50 556 (64.2)	333 216 (56.9)
Race/ethnicity, No. (%)			
Non-Hispanic White	418 980 (82.7)	67 701 (86.0)	486 681 (83.2)
Non-Hispanic Black	44 365 (8.8)	5481 (7.0)	49 846 (8.5)
Hispanic	24 100 (4.8)	3374 (4.3)	27 474 (4.7)
Other Race/Ethnicity	19 065 (3.8)	2160 (2.7)	21 225 (3.6)
Clinical characteristics			
Has dementia, No. (%)	259 874 (51.3)	60 810 (77.3)	320 684 (54.8)
Charlson Comorbidity Index, mean (SD)	5.35 (3.2)	4.58 (2.9)	5.25 (3.2)
Frailty index,^b^ mean (SD)	0.21 (0.1)	0.23 (0.1)	0.21 (0.1)
Frail, No. (%)	107 562 (21.2)	25 491 (32.4)	133 053 (22.7)
2+ Hospitalizations,^c^ No. (%)	146 130 (28.9)	20 378 (25.9)	166 508 (28.5)
Area-level characteristics, No. (%)			
Census region^d^			
North-East	94 223 (18.6)	16 422 (20.9)	110 645 (18.9)
Midwest	117 693 (23.2)	17 894 (22.7)	135 587 (23.2)
South	209 210 (41.3)	31 807 (40.4)	241 017 (41.2)
West	85 384 (16.9)	12 593 (16.0)	97 977 (16.7)
RUCA rurality^e^	71 912 (14.2)	3632 (4.6)	75 544 (12.9)
Social Deprivation Index,^f^ median (IQR)	48.00 (25-71)	38 (25-71)	47.00 (24-70)
Less disadvantaged, No. (%)	162 216 (32.0)	33 814 (43.0)	196 030 (33.5)
Moderately disadvantaged, No. (%)	173 375 (34.2)	23 960 (30.4)	197 335 (33.7)
Severely disadvantaged, No. (%)	170 919 (33.7)	20 942 (26.6)	191 861 (32.8)
HBMC provider density,^g^ median (IQR)	2.87 (0-6.25)	5.44 (2.18-8.81)	3.21 (0-6.81)
Less dense, No. (%)	179 272 (35.4)	15 709 (20.0)	194 981 (33.3)
Moderately dense, No. (%)	172 043 (34.0)	22 561 (28.7)	194 604 (33.3)
Highly dense, No. (%)	155 195 (30.6)	40 446 (51.4)	195 641 (33.4)

Abbreviations: HBMC, home-based medical care; RUCA, Rural-Urban Commuting Area.

^a^The sample represents patients receiving Medicare fee-for-service who died in 2019; we examined use of HBMC in the 2 years prior to death.

^b^The Kim Frailty Index (KFI) score was used to determine frailty (nonfrail = KFI < 0.25; frail = KFI ≥ 0.25).

^c^Two or more hospital encounters from 366 to 730 days before death was taken into account.

^d^Census region divided into 4 categories: North-East: New Jersey, New York, Pennsylvania, Connecticut, Maine, Massachusetts, New Hampshire, Rhode Island, Vermont; Midwest: Illinois, Indiana, Michigan, Ohio, Wisconsin, Iowa, Kansas, Minnesota, Missouri, Nebraska, North Dakota, South Dakota; South: Delaware, Washington, DC, Florida, Georgia, Maryland, North Carolina, South Carolina, Virginia, West Virginia, Alabama, Kentucky, Mississippi, Tennessee, Arkansas, Louisiana, Oklahoma, Texas; West: Arizona, Colorado, Idaho, Montana, New Mexico, Nevada, Utah, Wyoming, Alaska, California, Hawaii, Oregon, Washington.

^e^RUCA used to classify rural (7–10 and 99 on primary RUCA variable) or urban (1–6 on primary RUCA variable includes Metro + Micro).

^f^Social Deprivation Index score was divided into tertiles with score range as follows: less disadvantaged (tertile I: 1–28), moderately disadvantaged (tertile II: 29–62), and severely disadvantaged (tertile III: 63–100).

^g^HBMC provider density per 10 000 population (based on data from previous research work) was divided into tertiles with the following scores in each category, highly dense (tertile III: 6.94–43.06), moderately dense (tertile II: 3.53–6.93), and less dense (tertile I: 0.3–3.52).

Nearly two-thirds of HBMC use occurred in congregate care settings (64.1%; eg, assisted living). Among HBMC users, 70.8% used any hospice, which was significantly higher than the use of hospice among those who did not use HBMC (59.7%). Similarly, among hospice users, HBMC users had significantly higher median length of stay (28 days) than non–HBMC users (20 days) (Table 1). As shown in Figure 1, hospice length of stay varied by HBMC and dementia status. Individuals without dementia and no HBMC had the highest percentage of very short hospice stays (18.7%: 1–3 days; 33.5%: 4–17 days). In comparison, individuals with dementia who used HBMC were least likely to have short hospice duration (10.7%; 1–3 days). Conversely, nearly 20% of individuals with dementia who did not use HBMC had hospice stays of more than 180 days compared with only 12.9% of individuals with dementia who did use HBMC, suggesting that HBMC mitigated against very short and very long hospice stays. Appendix Tables S1 and 2 depict HBMC and hospice use and duration (respectively) stratified by dementia.

**Figure 1.**
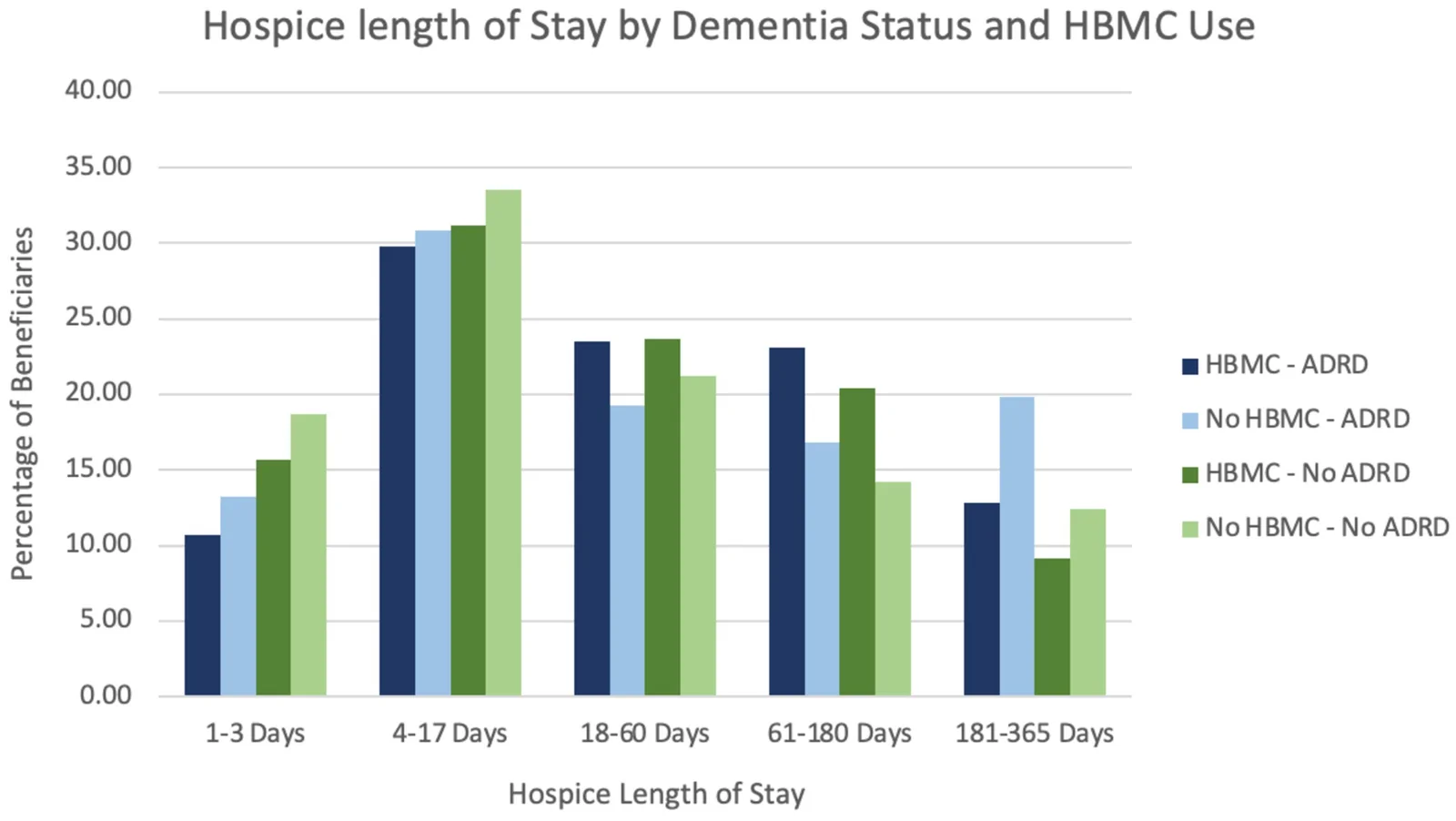
Hospice length of stay by dementia and HBMC use status (unweighted). Abbreviations: ADRD, Alzheimer's disease and related dementias; HBMC, home-based medical care.

After applying IPW, the balance of observable characteristics between HBMC exposure groups was considerably improved: the SMD in the sample between HBMC users and nonusers was below 10% for all variables considered (Appendix Figure S2). In weighted logistic regression examining hospice use, HBMC users had 1.34 greater odds of hospice use (odds ratio [OR]: 1.34; 95% CI: 1.33–1.35) (Table 2). Findings did not vary when stratified by dementia status (Appendix Table S1) and the interaction term was not significant (*P* = .28).

**Table 2.** Association between HBMC and hospice use.

	Adjusted OR (95% CI)
No.	585 226
HBMC use	1.34 (1.33, 1.35)
ADRD	1.72 (1.71, 1.74)
Age	1.02 (1.02, 1.02)
Sex, female (ref: male)	1.15 (1.15, 1.16)
Race (ref: non-Hispanic White)	
Hispanic	0.76 (0.75, 0.78)
Non-Hispanic Black	0.53 (0.52, 0.54)
Other	0.67 (0.66, 0.68)
Charlson Comorbidity Index	1.04 (1.04, 1.04)
Frail	0.88 (0.88, 0.89)
≥ 2 Hospitalizations	0.83 (0.82, 0.84)
Rural (ref: urban)	0.83 (0.82, 0.84)
Census region (ref: South)	
Midwest	0.98 (0.97, 0.99)
North-East	0.53 (0.52, 0.54)
West	0.86 (0.85, 0.87)
Social Deprivation Index^a^	
Moderately disadvantaged	0.84 (0.83, 0.85)
Severely disadvantaged	0.69 (0.68, 0.70)
HBMC provider density^b^	
Less dense	1.04 (1.03, 1.05)
Moderately dense	1.15 (1.14, 1.16)

Abbreviations: ADRD, Alzheimer's disease and related dementias; HBMC, home-based medical care; IPW, inverse probability weighting; OR, odds ratio; ref, reference.

ORs with 95% CI tested whether HBMC use predicted use of hospice in IPW logistic regression model adjusting for dementia status, age at death, sex, race/ethnicity, Charlson Comorbidity Index, frailty, multiple hospitalizations, census region, urban/rural classification, Social Deprivation Index, and, indicator of HBMC provider density.

^a^Ref = less disadvantaged.

^b^Ref = highly dense (tertile III: 6.94–43.06).

Hospice length of stay was significantly different for HBMC users and nonusers, such that decedents who used HBMC were less likely to use hospice 1 to 3 days or more than 180 days. Home-based medical care users in general had 34% reduced odds of having a hospice stay of 1 to 3 days (vs 18–60 days) compared with non–HBMC users (Appendix Table S3). Similarly, they were half as likely to have hospice length of stay greater than 180 days (vs 18–60 days) compared with non–HBMC users. The interaction of dementia and HBMC was significant for all levels of hospice duration (*P* < .001) and adjusted ORs and 95% CIs are also reported stratified by dementia status (Appendix Table S3). As shown in Figure 2, among individuals with dementia, HBMC users had a 9.4% predicted probability of hospice stays over 180 days, representing a 6.4 percentage point reduction compared with non–HBMC users (15.8%). Non–HBMC users without dementia had the highest predicted probability of hospice stays of 1 to 3 days (20.5%), followed by HBMC users without dementia (17.0%), followed by non–HBMC users with dementia (15.6%). The HBMC users with dementia had the lowest probability of having hospice stays of 1 to 3 days (12.6%).

**Figure 2.**
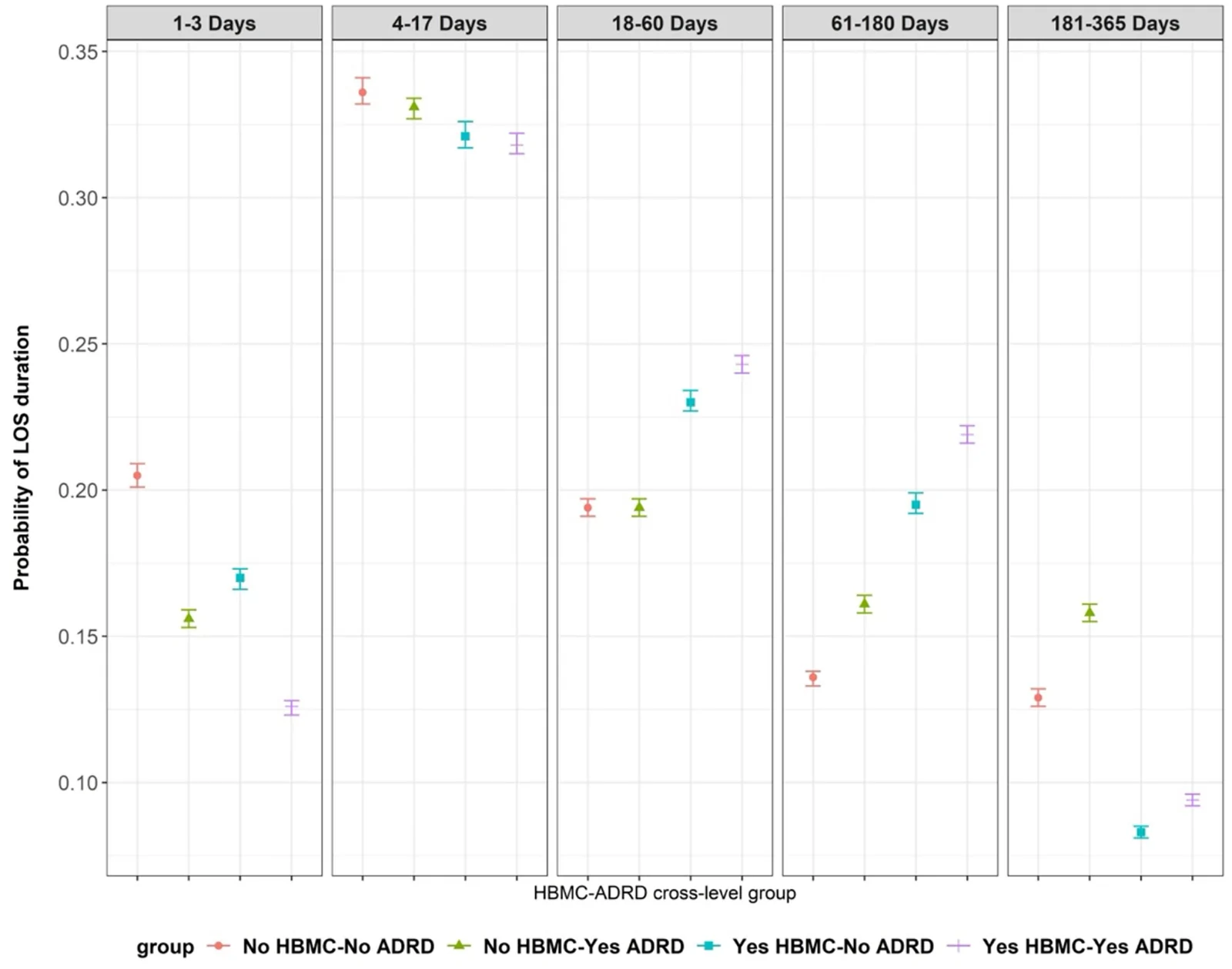
Probability of hospice duration by HBMC use and dementia status. Results based on a multinomial regression model including the interaction term, HBMC*ADRD. The marginal effects were estimated at representative values of age = 84.27 years, sex = male, Race = White, Charlson Comorbidity Index = 5.18, nonfrail, no hospital encounter, urban, census region = South, less disadvantaged SDI, and high-density HBMC provider. Data were propensity weighted to balance patient characteristics between those with and without HBMC use. Hospice duration was divided into 5 levels: 1–3 days, 4–17 days, 61–180 days, 181–365 days, with 18–60 days used as the reference level. Abbreviations: ADRD, Alzheimer's disease and related dementias; HBMC, home-based medical care; LOS, length of stay; SDI, Social Deprivation Index.

## Discussion

We found a strong association between HBMC use among those who were HBMC eligible and used hospice, regardless of dementia status. Although we hypothesized that HBMC would be especially important for facilitating hospice use for persons with dementia, we did not see evidence of an interaction when we examined hospice use overall. Among hospice users, we found that HBMC was associated with more timely hospice use. We also found that HBMC mitigated against the significant variation in length of stay often seen in hospice for those with dementia: HBMC was more protective against exceedingly short or long hospice stays for those with dementia.

Referral to timely hospice care is a critical component of high-quality end-of-life care. Hospice use improves quality of end-of-life care (higher family ratings of excellent care, better symptom management, fewer setting changes at the end of life) for decedents, including those with dementia, living in the community.^13^ Yet, there remain wide-ranging disparities in use of hospice care, including by race, diagnosis, and socioeconomic status.^34,35^ Interventions to increase timely hospice referral for all populations, including those with complex illness, are needed. Although there has been limited research about hospice use among HBMC users, qualitative work on hospice referral in skilled home health specifically demonstrates that clinicians working in the home recognize major regulatory, structural, and attitudinal barriers to hospice referral for home health patients, including difficulties with prognostication (especially in dementia) and family hesitance due to beliefs about hospice meaning “giving up.”^36,37^ Our finding that HBMC users with dementia had fewer stays greater than 180 days suggests that HBMC providers may have better insights on prognosis, which is notoriously challenging in this population.

There are several reasons why HBMC may be associated with more prognosis-aligned hospice use. The longitudinal relationships between clinicians, patients, and families can contribute to deeper provider knowledge of health needs so that HBMC clinicians may have earlier recognition of clinical decline.^38,39^ Similarly, longer relationships may lead to greater trust between providers, patients, and families, which, in turn, may foster conversations about the future and what treatments for patients remain aligned with their care. Because homebound patients often have a relatively worse prognosis, HBMC providers may have developed expertise in goals of care conversations, palliative care or advance care planning, and feel more confident initiating these conversations in the home setting.^40^ Furthermore, HBMC providers may have greater familiarity with hospice eligibility criteria and established connections to hospice agencies, which can facilitate timely referral.^41-45^

Importantly, as HBMC increasingly occurs in congregate settings such as assisted-living facilities, more work will be needed to understand the relationship between hospice use and HBMC within the context of noninstitutionalized care settings. Furthermore, the role of co-ownership, for-profit status, and the increasing role of private equity investment should also be considered.^46^ In a post hoc unadjusted analysis we found that HBMC users in congregate settings were, in fact, more likely to use hospice (75.9%) than HBMC users in home settings (61.6%). Similarly, among hospice users, those living in congregate settings with HBMC had higher median length of stays (31 days) compared with those who received HBMC at home (23 days). Future work will be needed to understand this relationship further, particularly among those with dementia. Although this is the first population-based study focused on HBMC and hospice use, our findings are consistent with the limited research on the positive role that home-based clinical services such as skilled home health care can have on facilitating hospice use.

The use of HBMC is growing in the United States, particularly among those living in assisted-living facilities^10^; yet, only a small proportion of Medicare beneficiaries (13.4%) who qualified for HBMC according to validated criteria use or have access to HBMC in their last years of life. Our study adds to the growing evidence base that HBMC can promote access to quality care for seriously ill populations^35^ and that HBMC successfully serves the most frail and at-risk patients among those with serious illness. Further examination of how HBMC programs interface with hospice and home health is warranted to better maximize this support process, particularly in the assisted-living setting. Similar to other studies, we also found social disadvantage to be a driver of nonenrollment and short length of stay in hospice. This suggests a continued need to study not just service delivery but the structural factors that create inequity in hospice use and how HBMC may contribute to achieving more equitable access to needed hospice care.

This study has several notable strengths. By leveraging 100% Medicare claims data, it enables a comprehensive examination of HBMC, a relatively uncommon model of care delivery in the general Medicare population. To our knowledge, this is the first analysis to specifically evaluate the role of HBMC in hospice use, addressing a major gap in the literature. The study also provides insights into differential patterns of care among those with dementia. Finally, we simultaneously considered access to HBMC by including a measure of HBMC density in analyses.

## Conclusion

This study provides further evidence of the benefits of HBMC by demonstrating increased and more timely hospice use, particularly among those patients with dementia. As more older adults age in the community and hospice services expand to support patients with complex illnesses such as dementia, HBMC may offer a valuable strategy for addressing ongoing challenges related to prognosis uncertainty and the complexities of hospice eligibility requirements.

## Supplementary Material

qxag251_Supplementary_Data

## Data Availability

Data are not available due to access restrictions.
