# Supplementary material for: Closing the hospice gap: the value of home-based medical care
Source: Health Aff Sch. 2026 Sep 17;4(10):qxag251. doi: 10.1093/haschl/qxag251 (PMC13653336; doi:10.1093/haschl/qxag251)
Supplement: Supplementary file 1 [file qxag251_supplementary_data.zip › HBMC-Hospice Appendix v2.docx]

**Appendix**

**Tables**

**Appendix Table 1: HBMC and Hospice Use regression analysis stratified by dementia**

|  | **ADRD cohort** | **NO ADRD Cohort** |
| --- | --- | --- |
|  | N = 320,684 | N = 264,542 |
| **HBMC Use** | **1.34 (1.32, 1.35)** | **1.34 (1.32, 1.35)** |
| ADRD |  |  |
| Age | 1.01 (1.01, 1.01) | 1.02 (1.02, 1.02) |
| Sex, Female (ref- male) | 1.1 (1.09, 1.12) | 1.20 (1.19, 1.21) |
| Race (ref- NH White) |  |  |
| Hispanic | 0.77 (0.75, 0.79) | 0.79 (0.77, 0.81) |
| NH Black | 0.61 (0.60, 0.62) | 0.47 (0.46, 0.48) |
| Other | 0.63 (0.61, 0.65) | 0.73 (0.71, 0.75) |
| Charlson Comorbidity Index | 0.97 (0.97, 0.97) | 1.09 (1.09, 1.09) |
| Frail (KFI>=0.25) (ref- Non-Frail) | 1.04 (1.03, 1.06) | 0.79 (0.77, 0.79) |
| >= 2 Hospitalizations | 0.87 (0.85, 0.88) | 0.86 (0.85, 0.87) |
| Rural (ref-urban) | 0.78 (0.76, 0.79) | 0.9 (0.88, 0.92) |
| Census Region (ref-South) |  |  |
| Mid-West | 1.02 (1.01, 1.04) | 0.94 (0.93, 0.96) |
| North-East | 0.50 (0.49, 0.51) | 0.56 (0.55, 0.57) |
| West | 0.92 (0.90, 0.93) | 0.81 (0.79, 0.82) |
| Social Deprivation Index (ref- Less Disadvantaged (Tertile I: 1-28)) | | |
| Mod Disadvantaged (T II: 29-62) | 0.83 (0.82, 0.84) | 0.85 (0.84, 0.87) |
| Severely Disadvantaged (T III: 63-100) | 0.68 (0.67, 0.69) | 0.72 (0.71, 0.73) |
| HBMC provider density/10k (ref- Highly Dense (Tertile III: 6.94-43.06) | | |
| Less Dense (T I: 0.3-3.52) | 1.02 (1.00, 1.04) | 1.04 (1.02, 1.06) |
| Mod Dense (T II: 3.53-6.93) | 1.11 (1.10, 1.12) | 1.18 (1.17, 1.20) |

**Appendix Table 2: HBMC and Hospice Duration regression analysis stratified by dementia**

|  | Hospice Level  (Ref- 18-60 days) | ADRD cohort | NO ADRD Cohort |
| --- | --- | --- | --- |
|  |  | N = 216,098 | N = 141,153 |
| HBMC Use | 1-3 days | 0.64 (0.63, 0.66) | 0.70 (0.69, 0.72) |
|  | 4-17 days | 0.77 (0.76, 0.78) | 0.81 (0.79, 0.83) |
|  | 61-180 days | 1.08 (1.06, 1.10) | 1.22 (1.19, 1.24) |
|  | 181- 365 days | 0.47 (0.46, 0.48) | 0.54 (0.52, 0.55) |
| Age | 1-3 days | 1.01 (1.01, 1.01) | 1.00 (1.00, 1.00) |
|  | 4-17 days | 1.01 (1.01, 1.01) | 1.00 (1.00, 1.00) |
|  | 61-180 days | 1.01 (1.01, 1.01) | 1.01 (1.01, 1.01) |
|  | 181- 365 days | 1.02 (1.02, 1.02) | 1.05 (1.04, 1.05) |
| Sex (ref- male) | 1-3 days | 0.83 (0.81, 0.85) | 0.88 (0.86, 0.90) |
|  | 4-17 days | 0.93 (0.91, 0.94) | 0.93 (0.91, 0.95) |
|  | 61-180 days | 1.06 (1.04, 1.08) | 1.00 (0.98, 1.03) |
|  | 181- 365 days | 1.24 (1.21, 1.27) | 1.08 (1.05, 1.11) |
| Race (ref- NH White) |  |  |  |
| Hispanic | 1-3 days | 1.07 (1.02, 1.13) | 0.96 (0.90, 1.02) |
|  | 4-17 days | 1.05 (1.01, 1.10) | 1.07 (1.01, 1.13) |
|  | 61-180 days | 1.03 (0.98, 1.07) | 1.05 (0.98, 1.11) |
|  | 181- 365 days | 0.99 (0.95, 1.05) | 1.16 (1.08, 1.25) |
| NH Black | 1-3 days | 0.80 (0.76, 0.83) | 0.94 (0.89, 0.99) |
|  | 4-17 days | 1.00 (0.97, 1.03) | 1.04 (1.00, 1.09) |
|  | 61-180 days | 0.82 (0.79, 0.86) | 0.99 (0.94, 1.04) |
|  | 181- 365 days | 0.81 (0.78, 0.84) | 1.05 (0.99, 1.12) |
| Other | 1-3 days | 1.23 (1.15, 1.31) | 0.84 (0.78, 0.89) |
|  | 4-17 days | 1.10 (1.04, 1.16) | 0.88 (0.83, 0.92) |
|  | 61-180 days | 1.02 (0.96, 1.08) | 1.09 (1.02, 1.16) |
|  | 181- 365 days | 0.97 (0.91, 1.03) | 0.92 (0.85, 0.99) |
| Charlson Comorbidity Index | 1-3 days | 1.03 (1.03, 1.04) | 0.97 (0.97, 0.97) |
|  | 4-17 days | 1.01 (1.00, 1.01) | 0.99 (0.99, 0.99) |
|  | 61-180 days | 1.00 (0.99, 1.00) | 0.98 (0.98, 0.99) |
|  | 181- 365 days | 0.93 (0.92, 0.93) | 0.94 (0.93, 0.94) |
| Frail (KFI>=0.25) | 1-3 days | 1.01 (0.98, 1.04) | 1.23 (1.19, 1.28) |
| Ref- Non-Frail | 4-17 days | 1.03 (1.01, 1.05) | 1.06 (1.03, 1.09) |
|  | 61-180 days | 1.18 (1.16, 1.21) | 1.23 (1.19, 1.27) |
|  | 181- 365 days | 1.54 (1.50, 1.58) | 1.65 (1.59, 1.71) |
| >= 2 Hospitalizations | 1-3 days | 1.12 (1.09, 1.15) | 1.01 (1.06, 1.12) |
|  | 4-17 days | 1.00 (0.98, 1.02) | 1.02 (1.00, 1.04) |
|  | 61-180 days | 0.98 (0.95, 1.00) | 0.94 (0.92, 0.96) |
|  | 181- 365 days | 1.03 (1.00, 1.06) | 1.04 (1.01, 1.07) |
| Rural (ref-urban) | 1-3 days | 0.94 (0.91, 0.98) | 1.03 (0.99, 1.07) |
|  | 4-17 days | 0.99 (0.96, 1.02) | 0.95 (0.92, 0.98) |
|  | 61-180 days | 0.99 (0.96, 1.02) | 1.02 (0.98, 1.06) |
|  | 181- 365 days | 0.91 (0.88, 0.94) | 0.97 (0.93, 1.02) |
| Census Region (ref-South) | |  |  |
| Mid-West | 1-3 days | 1.01 (0.99, 1.04) | 0.91 (0.88, 0.94) |
|  | 4-17 days | 1.01 (0.99, 1.03) | 1.02 (0.99, 1.04) |
|  | 61-180 days | 0.97 (0.95, 1.00) | 0.90 (0.87, 0.93) |
|  | 181- 365 days | 0.89 (0.87, 0.91) | 0.80 (0.77, 0.83) |
| North-East | 1-3 days | 1.14 (1.10, 1.18) | 0.87 (0.84, 0.90) |
|  | 4-17 days | 1.05 (1.02, 1.08) | 0.98 (0.95, 1.00) |
|  | 61-180 days | 0.93 (0.91, 0.95) | 0.85 (0.82, 0.88) |
|  | 181- 365 days | 0.74 (0.72, 0.77) | 0.62 (0.60, 0.65) |
| West | 1-3 days | 0.95 (0.92, 0.99) | 0.82 (0.79, 0.85) |
|  | 4-17 days | 1.00 (0.98, 1.03) | 0.99 (0.96, 1.02) |
|  | 61-180 days | 1.04 (1.01, 1.07) | 0.95 (0.91, 0.99) |
|  | 181- 365 days | 1.03 (1.00, 1.06) | 0.94 (0.90, 0.98) |
| Social Deprivation Index [Ref- Less Disadvantaged (Tertile I: 1-28)] | | | |
| Mod Disadvantaged (Tertile II: 29-62) | 1-3 days | 1.07 (1.04, 1.1) | 0.94 (0.91, 0.97) |
|  | 4-17 days | 1.03 (1.10, 1.05) | 1.01 (0.99, 1.04) |
|  | 61-180 days | 1.00 (0.98, 1.03) | 1.00 (0.97, 1.03) |
|  | 181- 365 days | 0.97 (0.95, 0.99) | 1.03 (0.99, 1.06) |
| Severely Disadvantaged (Tertile III: 63-100) | 1-3 days | 1.04 (1.01, 1.07) | 1.06 (1.03, 1.09) |
|  | 4-17 days | 1.02 (1.00, 1.05) | 1.04 (1.01, 1.06) |
|  | 61-180 days | 0.96 (0.94, 0.99) | 1.08 (1.04, 1.11) |
|  | 181- 365 days | 0.94 (0.91, 0.96) | 1.10 (1.07, 1.15) |
| HBMC provider density per 10k bene [Ref- Highly Dense (Tertile III: 6.94-43.06)] | | | |
| Less Dense (Tertile I: 0.3-3.52) | 1-3 days | 0.84 (0.81, 0.86) | 0.80 (0.77, 0.83) |
|  | 4-17 days | 0.90 (0.87, 0.92) | 0.90 (0.87, 0.92) |
|  | 61-180 days | 1.03 (1.01, 1.06) | 1.03 (0.99, 1.07) |
|  | 181- 365 days | 0.95 (0.92, 0.98) | 1.06 (1.01, 1.10) |
| Mod Dense (Tertile II: 3.53-6.93) | 1-3 days | 0.88 (0.86, 0.90) | 0.84 (0.82, 0.87) |
|  | 4-17 days | 0.95 (0.93, 0.97) | 0.88 (0.86, 0.90) |
|  | 61-180 days | 1.02 (1.00, 1.05) | 0.97 (0.94, 0.99) |
|  | 181- 365 days | 0.97 (0.95, 1.00) | 0.95 (0.92, 0.99) |

Notes: Adjusted odds ratios for hospice length of stay stratified by ADRD status, results from multinomial logistic regression with data using propensity weighting to balance patient characteristics between those with and without HBMC use.

**Appendix Table 3.** **Association between HBMC use and hospice length of stay, stratified by dementia status**

| **Hospice Duration Category** | **Model 1: Total  OR (95% CI)** | **Model 2: ADRD**  **OR (95% CI)** | **Model 3: NO ADRD**  **OR (95% CI)** |
| --- | --- | --- | --- |
|  | N = 358,061 | N = 216,098 | N = 141,153 |
| **1-3 days** | 0.66 (0.65, 0.68) | 0.64 (0.63, 0.66) | 0.70 (0.69, 0.72) |
| **4-17 days** | 0.78 (0.77, 0.79) | 0.77 (0.76, 0.78) | 0.81 (0.79, 0.83) |
| **61-180 days** | 1.13 (1.12, 1.15) | 1.08 (1.06, 1.10) | 1.22 (1.19, 1.24) |
| **181- 365 days** | 0.50 (0.49, 0.51) | 0.47 (0.46, 0.48) | 0.54 (0.52, 0.55) |

Notes.

HBMC=home-based medical care

Results from multinomial logistic regression with data using IPW to balance patient characteristics between those with and without HBMC use. Hospice length of stay reference group= 18-60 days.

Model 1 adjusted for dementia status, age at death, sex, race/ethnicity, Charlson Comorbidity Index, frailty, multiple hospitalizations, census region, urban/rural classification, Social Deprivation Index, and HBMC Provider density.

Models 2 and 3 adjusted for all covariates as model 1 minus dementia.

**Figures**

**Appendix Figure 1:** **Study Sample Derivation**

**Appendix Figure 2: Standardized Mean Differences of Inverse Probability Weighted characteristics of HBMC vs non- HBMC users**
